# Cultural intelligence and sales performance in online insurance marketing: evidence from a Chinese insurance firm

**DOI:** 10.1057/s41599-023-01623-z

**Published:** 2023-03-24

**Authors:** Guochen Pan, Mengqi Liu, Lu-Ming Tseng, Zhixiang Geng

**Affiliations:** 1 Economics and Management School, Wuhan University, Wuhan, China; 2 College of Finance, Feng Chia University, Taichung, Taiwan

**Keywords:** Business and management, Business and management, Cultural and media studies, Sociology

## Abstract

The cultural clash between customers and salespeople in online marketing is observed as a barrier to promoting sales performance.The capability of handling cultural difference, or cultural intelligence (CQ), is therefore essential for salespeople. With data collected through questionnaires from a Chinese digital insurance brokerage firm, the impact of salespeople’s CQ on sales performance is examined with the partial least squares structural equation model (PLS-SEM) method. It is identified that CQ serves as a positive moderating variable in the relationship between customer orientation and sales performance, as well as a partial mediating variable in the relationship between perceived organizational support and sales performance of the online insurance salespeople.

## Introduction

When the Internet makes the world smaller, cultural clashes become more prominent. If marketing activities are limited to small geographical areas within a country, it is reasonable to expect that the cultural gap rarely exists between salespeople and customers. Hence the salespeople’s capability to handle the cultural difference is not essential. However, things would be different when the marketing activities are expanded to a broad geographical area with heterogeneous folk traditions and cultures. Consider China, where cultural diversity is prominent despite the fact that the majority of the population is of Han nationality. First, there exists an array of religions within mainland China. The primary religion of Confucianism coexists with Taoism, Han Buddhism, Tibetan Buddhism, Islamism, Christianity, etc. Second, the national culture of China is a medley of the mainstream Han culture and minority cultures. Each of the 56 ethnic groups has their own unique tradition, including their own dance, music, life etiquette, festival, ceremony, and so on. Third, the diversity of regional culture is enormous, which can be attributed to vast territory of China. Scholars have found a connection between economic development and culture types in different regions of China (Liu, 2020). Regional culture may have a more direct and specific influence on marketing (Cho et al., 2010).

With the rising number of Internet users and the subsequent surge of online marketing, online marketing salespeople must deal with geographically distant consumers with diverse cultural backgrounds, giving rise to significant cultural issues that need addressing. Pidduck et al. (2022) declare that cultural intelligence (CQ) is an increasingly valuable asset for managers, employees, entrepreneurs, and their organizations. Delpechitre and Baker (2017) believe that identifying and confirming the influence brought by cultural differences can help salespeople adapt their sales methods to conform to customers’ distinct cultural backgrounds and values. Chen and Gabrenya (2021) also propose that organizations, which put their employees in cross-cultural environments should be aware of the employees’ cross-cultural competence (3c). Even though these literature generally advise that firms be aware of the adverse effect caused by cultural differences, the impact of cultural difference and the role of CQ in online marketing have rarely been studied yet (Ott and Michailova, 2018).

It is interesting to look into the insurance industry, which is heavily dependent on marketing activities. Before the rise of the Internet, Chinese insurance firms generally recruited local residents as salespeople for their affiliates. Which in turn, trained these salespeople to sell insurance products to local customers. Now that both customers and salespeople are residents within the same cultural background, usually there is no cultural barrier obstructing communication. However, the widespread use of the Internet has changed the rules of the game. Through the Internet, salespeople can reach customers thousands of miles away with different cultural backgrounds. In recent years, a growing number of digital insurance firms and insurance intermediary organizations have emerged in China, providing insurance products across the country over the Internet. Meanwhile, the vast majority of the traditional insurance firms are trying to utilize online marketing as an alternative distribution channel. The proportion of premium income generated from online marketing in the total premium income has been increasing year by year. According to the data disclosed by the Insurance Association of China (IAC), insurance premium income from Internet channels reached 290 billion yuan in 2020, accounting for 6.4% of the total premium. The COVID-19 pandemic accelerated the growth of the online insurance market for businesses. For salespeople to meet the challenges posed by the growth in online sales, an important factor is how they understand and respond to cultural differences. However, the capacity of salespeople to adapt to different cultures, referred to as cultural intelligence (CQ), is presently grossly underestimated. CQ is defined by various studies as the capacity to engage effectively with other people from diverse cultural backgrounds. CQ is different from other types of intelligence, such as general intelligence and emotional intelligence. General intelligence often refers to logic-based, verbal, reasonable, and quantitative intelligence. Emotional intelligence refers to a person’s capacity to comprehend and communicate human emotions (Piqueras et al., 2019). Different from logic-based intelligence or emotion, the emphasis of CQ is on cross-cultural interactions, or the capability of people to adapt to a new culture when dealing with other people with different cultural backgrounds. The insurance industry is just a miniature of the national economy where the Internet promotes transactions but brings cultural conflicts as well. Unfortunately, cultural differences between the salesperson and the customer could be a barrier for online transactions. It is noted that salespeople with high CQ might be able to deal with the cultural difference and boost transactions. With data from the insurance industry, this article tries to examine the impact of online salespeople’s CQ on the sales performance. The research objective of this article is threefold. First, to discover the challenge brought by the broad use of the Internet from the perspective of culture. Secondly, to identify the role of salespeople’s CQ in online marketing performance. Third, to provide countermeasures for the online business firms. To our limited knowledge, this is the first research to study the role of CQ in online insurance marketing when the business model is fundamentally changed by the Internet, our research results could also extensive the management and operation of extensive online businesses.

## Literature review and hypotheses

### CQ and sales performance

Given the fact that CQ is a relatively new concept, there is no agreement on how to define it yet (various definitions can be found in literature, e.g., Earley and Ang (2003), Min et al. (2021). Earley and Ang (2003) are the first to propose the concept of CQ by defining CQ as the capability of people to collect and process information, make judgments and take corresponding effective measures to adapt to new culture in new cultural situations, a definition widely accepted henceforth. According to Earley and Ang (2003), the four-dimensional structure of CQ includes metacognitive CQ, cognitive CQ, motivational CQ, and behavioral CQ. Metacognitive CQ reflects the psychological processes used by individuals to obtain and understand cultural knowledge, including the understanding and control of cultural knowledge; cognitive CQ reflects the knowledge of norms, practices and conventions in different cultures obtained from education and personal experience, including the understanding of economic, legal and social systems of different cultures. People with high cognitive CQ can understand the similarities and differences of different cultures better. Motivational CQ reflects the capability to focus on learning and functioning in situations characterized by cultural differences; while behavioral CQ reflects the ability to exhibit appropriate verbal and nonverbal behaviors when interacting with people from different cultures.

Literature on the study of CQ largely focus on the influence of CQ on the adaptation to cross-cultural work changes (Li, 2020; Setti et al., 2022) or the performance of cross-cultural work (Charoensukmongkol and Pandey, 2020; Delpechitre and Baker, 2017). Nevertheless, as complained by Masrek et al. (2021), attention of CQ study should also be paid to national companies or organizations which are confronted with cultural conflicts. CQ is found to elicit positive attitudes and performance (Ramalu and Subramaniam, 2019). In this study concentrating on online insurance marketing in China, since the online insurance salespeople are exposed to diverse cultures, this article assumes that:

**H1**: The CQ of online insurance salespeople contains a positive impact on sales performance.

### Adaptive selling, CQ, and sales performance

Weitz et al. (1986) construct an analytical framework of adaptive selling behavior for the first time, and define adaptive selling behavior as “salespeople adjust sales behavior based on the nature of the perceived sales situation in the process of interacting with customers or among different customers.” They also propose that the impact of adaptive selling behavior on sales outcomes is moderated by the sales environment, knowledge, and skills of salespeople. Kwak et al. (2019) identify that adaptive selling strategy positively correlates with sales performance. Singh et al. (2017) state that sales teams must demonstrate strong capability of adaptive selling to achieve sales goals and lay the foundation for long-term profitable customer relationships. Other relevant studies have all confirmed that adaptive selling behavior has a significant positive effect on sales performance (Charoensukmongkol and Suthatorn, 2020; Zhou and Charoensukmongko, 2021).

It is discovered that there is a positive correlation between CQ and adaptive selling behavior (Charoensukmongkol, 2020; Zhou and Charoensukmongkol, 2022). The positive effect of CQ on adaptive selling may come from several sources. For instance, salespeople with high metacognitive CQ are prone to have the ability to think strategically, enabling them to adjust their sales skills to their customers more accurately (Sahin et al., 2014). Understanding customer needs with different cultural backgrounds and effectively adapting selling behavior to customer preference requires salespeople to have sufficient cultural knowledge. Therefore, salespeople with high cognitive CQ can understand cultural differences better, which is very important when engaging in adaptive selling behavior (Sahin et al., 2014). Salespeople with high motivational CQ will actively adjust their sales methods according to the customer’s cultural background, simplifying product information to be easily understood by the customer. Finally, considering that online insurance marketing requires a mutual understanding between salesperson and customer, the salespeople with higher behavioral CQ will demonstrate higher capability to display sales behaviors that are consistent with the cultural expectations of customers in other regions. As Presbitero and Attar (2018) point out, CQ can interact with other individual-level characteristics to yield desirable outcomes. Therefore, this article proposes the following hypotheses:

**H2**: Adaptive selling contains a positive impact on the sales performance of online insurance salespeople.

**H3**: CQ plays a positive role in moderating the relationship between adaptive selling and sales performance of online insurance salespeople.

### Customer orientation, CQ, and sales performance

Salespeople with more customer-oriented behaviors will be more willing to invest in understanding their customers, adopting behaviors that can strengthen customer relationships, or actively exchanging information with customers in an attempt to find solutions for customers’ concerns. Numerous results have revealed that customer orientation is positively correlated with sales performance and customer satisfaction (e.g., ELSamen et al., 2018; Lussier and Hartmann, 2016; Domi et al., 2020). Particularly, it is confirmed that customer orientation contributes to the performance of the insurance salespeople (Pousa et al., 2018), and employees of the banks as well (Koshksaray et al., 2020).

Consumers from different cultural backgrounds will have different service expectations and product preferences. Thus, the firms’ customer orientation strategy could be negatively affected by the cultural difference (Li et al., 2021), so be the salespeople’s performance. Salespeople with high metacognitive CQ tend to have information acquisition skills and consciously understand the customers in different regions before and during interactions. Salespeople with high cognitive CQ have a high degree of familiarity with the customs and special norms of different cultures and can appreciate the cultural characteristics of customers more quickly. Motivational CQ represents the fun and interest of interacting with customers with different cultural backgrounds (Ang et al., 2007), a salesperson with high motivational CQ has an intrinsic drive to actively understand the needs of their customers. Behavioral culture intelligence enables salespeople to better understand and satisfy customer needs in a comfortable way, thus positively affecting sales performance. Earley and Mosakowski (2004) believe that imitating the behavior of customers from different cultural backgrounds could be observed as being customer-oriented, while assimilating with the customers’ cultural background could be interpreted as highly respecting their culture. Therefore, the following hypotheses are presented in this article:

**H4**: Customer orientation contains a positive impact on the sales performance of online insurance salespeople.

**H5**: CQ plays a positive role in moderating the relationship between customer orientation and sales performance of online insurance salespeople.

### Perceived Organizational support, CQ, and sales performance

Perceived organizational support improves salespeople’s identification with the organization by satisfying the social-emotional needs of the salesperson. This can make salespeople feel obligated to promote the performance of the organization. Ridwan et al. (2020) find that perceived organizational support is positively correlated with outcomes that benefit both employees (e.g., job satisfaction, positive emotions) and the organization. Kurtessis et al. (2017) use the results of 558 studies to validate organizational support theory through meta-analysis and find that when perceived organizational support is higher, employees become more interested in their work, and employees’ commitment to the work increases.

Perceived organizational support is related to cultural adaptation. The more a salesperson has a sense of integration into the company, the more she/he identifies with the company, which helps to adapt to the environment better. Kraimer and Wayne (2004) find that organizational support is positively correlated with the adaptation of cross-cultural expatriates to interaction with residents. According to the contact theory, MacNab et al. (2012) confirm that as the fundamental requirement of effective contact, organizational or leadership support is positively correlated with the development of CQ. Lee and Kartika (2014) find that the level of organizational support not only enhances the positive impact of CQ on cross-cultural adjustment, but also strengthens the positive impact of cross-cultural adjustment on organizational performance. Therefore, this paper proposes the following research hypotheses:

**H6**: Perceived organizational support contains a positive impact on the sales performance of online insurance salespeople.

**H7**: CQ mediates the relationship between perceived organizational support and sales performance.

## Methods

### Conceptual research model

**Fig. 1:**
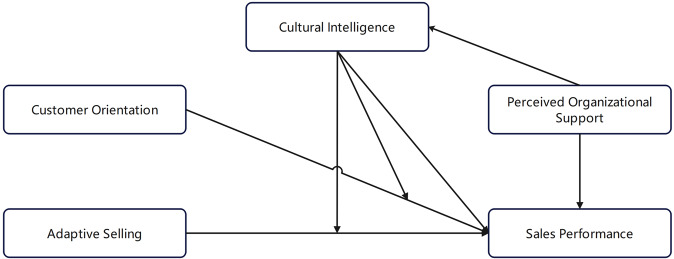
Conceptual research model.

According to the previous studies, factors influencing sales performance include adaptive selling (Charoensukmongkol and Suthatorn, 2020; Kwak et al., 2019; Singh et al., 2017; Zhou and Charoensukmongkol, 2021), customer orientation (Domi et al., 2020; ELSamen et al., 2018; Lussier and Hartmann, 2016; Koshksaray et al., 2020; Pousa et al., 2018), organizational support (Kurtessis et al., 2017; Ridwan et al., 2020), etc. This paper adds CQ variables to form a conceptual research model as shown in Fig. 1.

### Structural equation modeling

As there are latent variables in the model, the structural equation model (SEM) is superior to regression analysis and path analysis. SEM is employed to estimate a system of linear equations to test the fit of a hypothesized “causal” model as shown in Fig. 1. SEM comprises two sub-models, i.e., the measurement model which estimates relationships between the observed variables and the latent variable, and the structural model, which develops the relationships between the latent variables. The measurement model is written in matrix form as Eqs. (1) and (2):

1 $${{x}} = \Lambda _x\xi + \delta$$

2 $$y = \Lambda _y\eta + \varepsilon$$

where the *x* and *y* are observed indicators (or observed variables) for latent variables (such as CQ, customer orientation, etc.); the *ξ* and *η* are exogenous and endogenous latent variables, respectively; the Λ_x_ and Λ_y_ are factor loadings; and the *δ* and *ε* are error, or disturbance, term. The structural model can be written in matrix form as Eq. (3):

3 $$\eta = \alpha + B\eta + \Gamma \xi + \zeta$$

where *α* is a intercept term, *B* and *Γ* are the coefficients, ζ is the disturbance. ζ, *δ* and *ε* are assumed to be mutually uncorrelated. Note that control variable(s) is(are) not necessary in SEM, but with appropriate control variable(s) the evaluation of the impact of predictor variables on result variable will be more accurate. In our study, some basic information, namely gender, age, education level, managerial position, experience in industry, and number of professional certificates are included in Eqs. (1–3) as control variables.

When estimating SEM, two major methods are partial least square (PLS) method and linear structural relationships (LISREL) method. PLS method combines the statistical thought of principal component analysis and multiple regression. The main components are extracted from the observation variables of different potential variables to construct the regression model, and the parameters are estimated by adjusting the weights of the main components. LISREL method is based on the covariance structure, and model parameters are estimated by fitting the model covariance and sample covariance. LISREL uses maximum likelihood estimation, generalized least squares method or other methods to construct a fitting function between the covariance of model estimation and the covariance of sample data to obtain the parameter estimation that optimizes the value of the fitting function. The partial least squares structural equation model (PLS-SEM) is employed for current analysis. The reasons for adopting this method include: first, the PLS-SEM model combines principal component analysis and multiple regression analysis to maximize the explanatory power of endogenous variables, which is vital for our analysis; second, this model does not require the data to be normally distributed, which is a necessary feature because some data, such as age, are not normally distributed in our research; thirdly, PLS-SEM model is compatible with small sample sizes, which is suitable for our research where the sample size is less than 600. This article uses Smart PLS software for data processing. ANOVA analysis and other methods will be applied where appropriate as well.

### Data source

This article collects data through a questionnaire distributed to the insurance marketers of a medium-sized digital insurance brokerage company in China. There are six sections in the questionnaire (please see Supplementary Information). The first section covers the fundamental personal information and employment conditions of online insurance marketers. The second section is to measure the adaptive selling behavior of salespeople, and the questionnaire adopts the simplified ADAPTS scale of Robinson et al. (2002). The third section is to measure the degree of customer orientation of salespeople. The questionnaire is adapted from the SOCO scale of Saxe and Weitz (1982), which contains four forward measurement items and one reverse measurement item. The fourth section is a measurement of perceived organizational support. The questionnaire is adapted from the scale developed by Riggle et al. (2009), and five items are used to measure. The fifth section is to measure the CQ of the salespeople. The questionnaire is adapted from the SFCQ scale developed by Thomas et al. (2005). The measurement dimension consists of three aspects which are knowledge, skill, and cultural metacognition. The sixth section measures the job performance of the online insurance salespeople. The performance questionnaire is adapted from a scale developed by Williams and Anderson (1991), we narrow down the questions and use five items of them to measure. The results from the second to sixth section are on a Likert five-point system.

Considering the high acceptance of electronic questionnaires by online insurance marketers, the authors of this article use the “Questionnaire Star” platform to produce electronic questionnaires. With the support of the leaders of the surveyed companies, the questionnaires were distributed to more than 980 insurance marketers of the company through WeChat, a widely used social media in China, from April 21st to 25th in 2021, with a total collection of 601 questionnaires. After verification of the questionnaires, 29 were removed due to issues such as too short filling time duration, always selecting the same option, or abnormal basic information. Finally, 572 samples were accepted. The descriptive statistical results of basic information of the survey respondents are shown in Table 1:

**Table 1:** Descriptive statistics of respondents’ basic information.

Factor	Options	Count	Percent	Factor	Options	Count	Percent
Gender	Male	412	72%	Management	Management	56	10%
	Female	160	28%		Not Management	516	90%
Age	18–22	54	9%	Experience	0–12 months	232	41%
	23–26	235	41%		13–24 months	133	23%
	27–30	175	31%		25–36 months	103	18%
	31–33	88	15%		37–48 months	85	15%
	34–36	20	4%		Over 48 months	19	3%
Education	Graduate or higher	112	20%	Number of certificates	0	162	28%
	College	347	61%		1–2	398	70%
	Junior college	113	20%		3–5	12	2%

As can be seen from Table 1, the survey subjects are mostly male, young salespeople under the age of 30, and education level is concentrated around junior college, with the majority being non-managerial members. 28% of the salespeople have not obtained any work certifications or certificates pertaining to training related to work.

### Reliability analysis of questionnaire

Cronbach’s α coefficient is utilized to assess the reliability of the questionnaires. From Table 2, it can be seen that the overall Cronbach’s α coefficient of the questionnaire is 0.948, which is greater than 0.9, indicating the high reliability of the research data.

**Table 2:** Reliability statistics.

Cronbach’s *α*	Cronbach’s *α* based on standardized items	Items
0.942	0.948	572

Reliability analysis was conducted on the questionnaire results of each variable, and the corresponding standardized *α* values are: adaptive selling 0.91, customer orientation 0.85, perceived organizational support 0.89, CQ 0.94, and job performance 0.92. According to the standardized *α* value, the reliability of this questionnaire is high.

### Validity analysis of the questionnaire

In this paper, the validity of the questionnaire was studied with factor analysis. According to the results of Table 3, the coefficient of the KMO test is 0.947, and the significance is infinitely close to 0. The coefficient of the KMO test ranges from 0 to 1, and the closer it is to 1, the better the validity of the questionnaire will be. The value of 0.947 indicates that the questionnaire is relatively valid.

**Table 3:** KMO and Bartlett test.

Kaiser-Meyer-Olkin Measure of Sampling Adequacy		0.947
Bartlett’s Test of Sphericity	Approx. Chi-Square	12733.329
	df	435
	*P*-value	0

### Reliability and validity analysis of the measurement model

After adjusting the questionnaire entries, the measurement model was tested for reliability and validity. According to the estimated results of the measurement model parameters in Table 4, the load values of reliability factor in this paper are all greater than 0.7, which meets the requirements, indicating that the structure explains more than 50% of the index variance, and the index setting is reliable. Cronbach’s α values are all close to 0.9, and the combined reliability CR values are all greater than 0.7, which meets the requirements and indicates good internal consistency reliability. The average variance extraction values (AVE) of the indicators which evaluate the convergence validity are all greater than 0.5, which is in line with the criteria, indicating that each scale is highly correlated with other indicators of the same construct.

**Table 4:** Measurement model parameter estimation table.

	Items	Standard loadings	Cronbach’s *α*	Construct reliability (CR)	Average variance extracted (AVE)
Customer orientation	CO1	0.837	0.847	0.897	0.687
	CO2	0.863			
	CO3	0.847			
	CO5	0.765			
Adaptive selling	ASB1	0.863	0.910	0.933	0.736
	ASB2	0.809			
	ASB3	0.876			
	ASB4	0.890			
	ASB5	0.849			
Cultural intelligence	CQ1	0.716	0.944	0.952	0.667
	CQ2	0.749			
	CQ3	0.816			
	CQ4	0.828			
	CQ5	0.842			
	CQ6	0.865			
	CQ7	0.865			
	CQ8	0.851			
	CQ9	0.786			
	CQ10	0.834			
Organizational support	OS1	0.851	0.892	0.921	0.699
	OS2	0.871			
	OS3	0.860			
	OS4	0.803			
	OS5	0.791			
Performance	PM1	0.896	0.917	0.941	0.800
	PM2	0.884			
	PM3	0.917			
	PM4	0.881			

In addition, from the results in Table 5, it can be seen that a load of each construct factor is all higher than its cross load with other constructs, which conforms to the judgment criterion proposed by Fornell and Larcker (1981), and the model passes the discriminative validity test.

**Table 5:** Differential validity test table.

	CO	CQ	OS	ASB	PM
CO	0.829				
CQ	0.583	0.817			
OS	0.394	0.494	0.836		
ASB	0.541	0.692	0.381	0.858	
PM	0.501	0.626	0.495	0.646	0.894

## Empirical results

### Results of ANOVA analysis

The independent sample t-test and the one-way ANOVA method are used to test the six aspects which are gender, age, education level, managerial position, experience in industry and number of professional certificates (the table is omitted due to space limitation). It was found that differences in gender, education level, and status of managerial position do not affect adaptive selling, customer orientation, cultural intelligence, perceived organizational support, and sales performance. There are significant differences in organizational support for age. The perceived organizational support of 18 to 22 years old salespeople is higher than that of other groups, reflecting the importance of organizational support for fresh insurance salespeople. There are significant differences in adaptive selling, perceived organizational support, and sales performance in terms of working time.

Salespeople who have worked for less than a year have relatively poor adaptive selling levels, but the highest perceived organizational support. Salespeople who have been working for 3 to 4 years have the highest job performance. There are significant differences in customer orientation, cultural intelligence and organizational support in terms of the number of certificates. The larger the amount of certificates, the higher the customer orientation, cultural intelligence and perceived organizational support, indicating that there is a strong positive correlation between professional literacy and customer orientation and/or cultural intelligence.

### Collinearity diagnosis

As shown in Table 6, VIF values of the internal models are all less than 3, which means that the collinearity problem of each construct in the structural model is not serious.

**Table 6:** VIF analysis table.

	CO	CQ	OS	ASB	CQ*CO	CQ*ASB
Performance	1.944	2.391	1.386	2.069	2.112	1.817

### Structural model inspection

Two types of indicators can be used to evaluate the quality of the structural model. The first type of indicator evaluates the model’s explanatory ability, including the *R*^2^ value and the explanatory effect value *f*^2^; the second type of indicator evaluates the model’s predictive ability, including the significance of path coefficient and forecast correlation *Q*^2^. *R*^2^ reflects the amount of change in intrinsic constructs that can be explained by extrinsic constructs in the model. *R*^2^ is between 0 and 1. The higher the value, the higher the explanatory capability. It can be seen from Table 7 that the *R*^2^ of the independent variable to sales performance in this article is 0.535, which shows a strong explanatory capability. The explanatory effect value *f*^2^ measures the amount of change in the *R*^2^ value after deleting the specific exogenous variables in the model. It can be used to evaluate whether the exogenous variables have a significant explanatory capability to the intrinsic variables. According to Cohen’s *f*^2^ value evaluation criterion, aside from cultural intelligence having insignificant moderating effect on the relationship between adaptive selling and sales performance, all other extrinsic derivative variables have a significant explanatory capability. The *Q*^2^ of sales performance is significantly greater than 0, indicating that the structural model has a good predictive correlation.

**Table 7:** Interpretation and prediction capacity verification table.

Hypothesis	Relationship	*R*^2^	*f*^2^	*Q*^2^
H1	Adaptive Selling→Performance	0.535	0.132	0.422
H2	CQ*Adaptive Selling→Performance		0.006^#^	
H3	Customer Orientation→Performance		0.059	
H4	CQ*Customer Orientation→Performance		0.026	
H5	Cultural Intelligence→Performance		0.045	
H6	Organizational support→Performance		0.060	
H7	Organizational support→Cultural Intelligence	0.244	0.323	0.161

### Structural model regression results

**Fig. 2:**
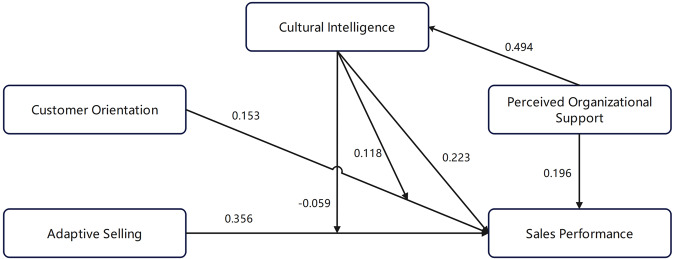
Path coefficients of the structural model.

Applying Smart PLS software to run the PLS-SEM model, the path coefficient value of the structural model is obtained (see Fig. 2). From the path coefficient verification table (Table 8), we can see that except for the insignificant moderating effect of CQ on adaptive selling (hypothesis H3), the rest of the path coefficients are all relatively significant, and the confidence interval does not cover 0 (except H5 path slightly covers 0).

**Table 8:** Path coefficient verification.

Hypothesis	Relationship	Std path coefficients	*t*-value	95%CILL	95%CIUL
H1	CQ → Performance	0.223^*^	4.459	0.121	0.318
H2	Adaptive Selling→Performance	0.356^*^	7.555	0.265	0.453
H3	CQ*Adaptive Selling→Performance	−0.059	0.750	−0.145	0.136
H4	Customer Orientation→Performance	0.153^*^	3.142	0.039	0.230
H5	CQ*Customer Orientation→Performance	0.118^*^	2.087	−0.034	0.183
H6	Organizational support→Performance	0.196^*^	5.297	0.125	0.270
H7	Organizational support→CQ	0.494^*^	13.301	0.424	0.566

*Means *p* < 0.05.

#### The impact of adaptive selling on sales performance and the moderating effect of CQ

The empirical results reveal that the salespeople’s adaptive selling behavior can significantly promote sales performance (H2). Salespeople with strong adaptive selling ability can flexibly switch selling methods according to different demand characteristics of customers etc., and pass on product information preferable to the customer, making it easier to achieve effective transactions. Our results are consistent with most of the previous literature (Charoensukmongkol and Suthatorn, 2020; Kwak et al., 2019; Singh et al., 2017; Zhou and Charoensukmongkol, 2021).

The role of CQ in regulating the relationship between adaptive selling and sales performance is not significant, which is not consistent with our hypothesis H3. We speculate that because the salespeople with high CQ are more receptive and inclusive of cultural differences, they may blur individual differences between customers, and lose the capability to accurately classify customers and adjust sales methods according to customer types, resulting in an insignificant role in regulating the relationship between adaptive selling and sales performance. According to Vlajcic et al. (2019), culturally intelligent employees are more creative in multicultural settings, supporting its weakened moderating role of promoting the effect of adaptive selling on sales performance.

#### The impact of customer orientation on sales performance and the moderating effect of CQ

Table 8 shows a significant positive correlation between the degree of customer orientation and sales performance (H4). Salespeople with a high degree of customer orientation are more committed to understanding and meeting the needs of customers, so they can easily be recognized by customers and their sales performance is relatively high. Our evidence is in line with the results of most literature about customer orientation and sales performance (ELSamen et al., 2018; Lussier and Hartmann, 2016; Domi et al., 2020; Koshksaray et al., 2020). Notablely, the results are similar to another study with data from insurance salespeople (Pousa et al., 2018).

Based on the path verification results, CQ positively regulates the relationship between customer orientation and sales performance (H5). In the case of online marketing, huge differences in cultural background between salespeople and their customers would objectively make it difficult for salespeople to fully understand the customer’s demands due to the difference in values, consumption concepts, communication methods, etc., resulting in low sales performance. Specifically, salespeople with high cognitive CQ can adapt to cultural differences faster and can better meet customer needs from the customer’s cultural background, those with high motivational CQ are more internally motivated to achieve work goals and actively consider appropriate sales strategies from the customer’s standpoint, and those with high behavioral CQ can show more customer-oriented behavior during the process of interacting with customers, dynamically catering to customers. In short, salespeople with high CQ can display better customer orientation and improve their sales performance. Our results are supported by Ramalu and Subramaniam (2019), where CQ is reckoned as a personal resource which facilitates work engagement, and employees with higher CQ can benefit from successful interactions with others by collecting more culturally diverse resources, and make it easier to turn customer orientation behavior into favorable results.

#### The impact of perceived organizational support on sales performance and the mediating role of CQ

The direct effect of perceived organizational support on sales performance is 0.196, the indirect effect through CQ is 0.494*0.223 = 0.110, and the total effect is 0.306. After considering the mediating effect of CQ, the confidence interval of the direct and indirect effects of the independent variable perceived organizational support on sales performance, the dependent variable does not contain 0 (H6 and H7), so CQ is identified to play a partially mediating role between the perceived organizational support of independent variable and the sales performance of the dependent variable (Table 9).

**Table 9:** Indirect effect verification.

Relationship	Std path coefficients	*t*-value	95%CILL	95%CIUL
Organizational support→CQ → Performance	0.110	4.168	0.059	0.162

Similar to Ridwan et al. (2020), perceived organizational support is found to be positively correlated with performance. Moreover, our study suggests that the perceived organizational support of salespeople influences sales performance partially through CQ. With the improvement of organizational support, better communication, and trust in the organization, the salespeople will be more willing to commit behaviors beneficial to the organization, thus generating higher motivational CQ, and being more active in coping with the cultural differences between themselves and customers, improving their sales performance.

## Conclusion

Based on the survey data from a digital insurance brokerage company that solely relies on online marketing, this study investigates the factors that affect online insurance salespeople’s performance, with particular focus on the impact of CQ. The following conclusions are obtained. First, customer orientation, adaptive selling, perceived organizational support, and CQ have a significant positive effect on sales performance, indicating that the performance of online salespeople is determined by various factors, but some new factors, such as CQ, are yet to be discovered when the condition of marketing changes. Second, higher CQ is conducive to the salespeople to amplify the role of customer orientation and promote sales performance. Vice versa. To be specific, insurance salespeople with high CQ are more likely to understand the customers’ concerns about risks in their position or situation, and provide appropriate insurance program(s) and relative services accordingly. Last but not the least, when employees are grateful to their leaders or feel indebted to the company, their CQ will be stimulated while processing business, for instance, they may become more willing to listen to the prospective insured and uncover their latent demand for insurance. Nowadays online insurance marketing is surging due to the wide use of the Internet, whereas cultural difference becomes a barrier to such a trend. CQ of the online salespeople is a key factor to address the situation. Findings of this article contain important practical significance for the operation and management of online insurance firms and reference value for the online marketing of other industries.

### Management enlightenment

At a time when online insurance marketing is booming, the findings of the study on CQ have the following implications for online insurance companies:

Firstly, while the use of online selling is still being explored, insurance companies should be aware that as Internet-driven marketing approaches evolve, factors impacting product marketing and service delivery experience dramatic changes, with cultural factors being one of them. Perhaps the development of the Internet will eventually fuse cultural boundaries, but that will be a long-term process. In the near future, the online insurance business will be confronted with the challenges brought by intercultural issues, it is vital to analyze its impact and formulate corresponding management strategies.

Secondly, because CQ not only contains a direct positive impact on sales performance, but also can positively adjust the relationship between some key factors (such as customer orientation) and sales performance, or play a mediating role, online insurance management should give priority to recruiting salespeople with relevant characteristics, or strengthen the training of existing salespeople to improve their CQ level.

Last but not the least, traditional techniques of organizational development may still be useful. Giving employees greater assistance and care will strengthen their perceptions of organizational support as well as their motivational and behavioral CQ, resulting in better sales performance.

### Limitations and future research suggestions

There are still some limitations and deficiencies in this study. To begin with, CQ is a relatively novel concept which is usually used to study issues about expatriates, and the relevant theories are not yet perfect. When we introduce this concept into the domestic fields of marketing, the theoretical construction might be immature. Secondly, this paper merely examines limited factors influencing insurance sales performance, which to some extent limits our exploration of the mechanism of CQ. Thirdly, this paper uses an electronic questionnaire to collect the data, which is somewhat different from the manual method and there may be a loss in data quality. Finally, the study in this paper is based on the assumption of cultural gaps, but it fails to accurately gauge the size of the gap. These deficiencies leave room for further exploration. Future research could attempt to study the role of CQ by classifying cultural types and measuring gaps more accurately. In addition, the influence of different dimensions of CQ on sales performance could be studied separately, and the relationship between CQ and other determinants of sales performance could also be discussed.

## Supplementary information

Supplementary information

## Data Availability

All data generated or analyzed during this study are included in this published article.
